# Dissociation of pulse wave velocity and aortic wall stiffness in diabetic db/db mice: The influence of blood pressure

**DOI:** 10.3389/fphys.2023.1154454

**Published:** 2023-03-24

**Authors:** Patricia E. McCallinhart, Yong Ung Lee, Avione Lee, Mircea Anghelescu, Jeffrey R. Tonniges, Ed Calomeni, Gunjan Agarwal, Joy Lincoln, Aaron J. Trask

**Affiliations:** ^1^ Abigail Wexner Research Institute, Nationwide Children’s Hospital, Columbus, OH, United States; ^2^ Center for Cardiovascular Research, Nationwide Children’s Hospital, Columbus, OH, United States; ^3^ Tissue Engineering Program and Surgical Research, Columbus, OH, United States; ^4^ Department of Biomedical Sciences, Philadelphia College of Osteopathic Medicine (PCOM), Suwanee, GA, United States; ^5^ Biophysics Graduate Program at The Ohio State University, Columbus, OH, United States; ^6^ Dorothy M. Davis Heart and Lung Research Institute, College of Medicine, The Ohio State University, Columbus, OH, United States; ^7^ Department of Pediatrics, College of Medicine, The Ohio State University, Columbus, OH, United States

**Keywords:** vascular stiffness, pulse wave velocity, hypertension, diabetes, metabolic syndrome, aortic biomechanics

## Abstract

**Introduction:** Vascular stiffness is a predictor of cardiovascular disease and pulse wave velocity (PWV) is the current standard for measuring *in vivo* vascular stiffness. Mean arterial pressure is the largest confounding variable to PWV; therefore, in this study we aimed to test the hypothesis that increased aortic PWV in type 2 diabetic mice is driven by increased blood pressure rather than vascular biomechanics.

**Methods and Results:** Using a combination of *in vivo* PWV and *ex vivo* pressure myography, our data demonstrate no difference in *ex vivo* passive mechanics, including outer diameter, inner diameter, compliance (Db/db: 0.0094 ± 0.0018 mm^2^/mmHg vs. db/db: 0.0080 ± 0.0008 mm^2^/mmHg, *p* > 0.05 at 100 mmHg), and incremental modulus (Db/db: 801.52 ± 135.87 kPa vs. db/db: 838.12 ± 44.90 kPa, *p* > 0.05 at 100 mmHg), in normal *versus* diabetic 16 week old mice. We further report no difference in basal or active aorta biomechanics in normal *versus* diabetic 16 week old mice. Finally, we show here that the increase in diabetic *in vivo* aortic pulse wave velocity at baseline was completely abolished when measured at equivalent pharmacologically-modulated blood pressures, indicating that the elevated PWV was attributed to the concomitant increase in blood pressure at baseline, and therefore “stiffness.”

**Conclusions:** Together, these animal model data suggest an intimate regulation of blood pressure during collection of pulse wave velocity when determining *in vivo* vascular stiffness. These data further indicate caution should be exerted when interpreting elevated PWV as the pure marker of vascular stiffness.

## Introduction

The stiffness of conduit arteries is an important prognostic indicator of vascular health and has been associated with hypertension, atherosclerosis, and diabetes (Mitchell et al., 2010; Townsend et al., 2015; Jia et al., 2018). The current clinical benchmark for detecting arterial stiffness is pulse wave velocity (PWV) as instituted by the American Heart Association Council for High Blood Pressure (Townsend et al., 2015; Jia et al., 2018). However, studies that date back to the 1920s, 1930s, 1940s, and 1950s have reported that PWV is influenced by factors including degree of the vessel’s smooth muscle cell contraction (Bramwell, 1937), vessel wall elasticity (Steele, 1937; Kraner et al., 1959), intra-arterial diastolic pressure (Steele, 1937), wall thickness (Sands, 1925; Steele, 1937; Kraner et al., 1959), vessel tortuosity (Sands, 1925; Turner and Hermann, 1927), diastolic diameter of the vessel (Kraner et al., 1959), density of the blood (Kraner et al., 1959), and blood flow velocity (Woolam et al., 1962).

Type 2 diabetes mellitus (T2DM) is a metabolic disorder characterized by the incidence of hyperglycemia and insulin resistance. T2DM is classified as a cardiovascular disease by the American Heart Association in part because two-thirds of diabetes-related deaths are due directly to heart disease (Grundy et al., 1999). The stiffness of conduit arteries is an important prognostic indicator of vascular health not only in diabetes, but also in hypertension and atherosclerosis (Mitchell et al., 2010; Townsend et al., 2015; Jia et al., 2018). The current clinical benchmark for detecting arterial stiffness is pulse wave velocity (PWV) as instituted by the American Heart Association Council on Hypertension (Jia et al., 2018; et al., 2015). It is well established that type 2 diabetic patients have elevated aortic stiffness, however, many of these studies rely solely on PWV as the stiffness determinant. (De Angelis et al., 2004; Swoboda et al., 2018).

Another complication and prognostic indicator of health is blood pressure; however, blood pressure in the T2DM db/db mouse model is somewhat unclear. One study showed a mild, but significant increase in blood pressure (systolic and diastolic) as measured by radiotelemetry in the absence of increased pulse pressure (Su et al., 2008). A previous study by our group did not observe increased blood pressure measured by radiotelemetry in 24 h averages in similar-aged mice (Katz et al., 2011). However, pilot studies in our laboratory suggested a link between increased blood pressure in this animal model under anesthesia and aortic pulse wave velocity. Further pilot data also suggested that passive aortic biomechanics were similar between control and db/db mice, suggesting a disconnect between increased pulse wave velocity (“stiffness” as measured clinically) and aortic tissue biomechanics. Therefore, we tested the hypothesis that increased aortic pulse wave velocity in db/db mice is driven by increased blood pressure, rather than passive aortic biomechanics. To test this hypothesis, we performed a comprehensive set of *ex vivo* aortic biomechanics experiments performed under basal, passive and active conditions, *in vivo* pulse wave velocity experiments performed measured at controlled blood pressures, and histological assessments of essential drivers of biomechanics in the extracellular matrix.

## Materials and methods

### Animals

Experiments were conducted on 16–17-week old male control, non-diabetic heterozygous Db/db and T2DM db/db mice that were obtained from The Jackson Laboratories. All mice were housed under a 12-h light/dark cycle at 22°C and 60% humidity and were allowed *ad libitum* access to standard low-fat laboratory chow and water. This study was conducted in accordance with the National Institutes of Health Guide for the Care and Use of Laboratory Animals, and it was approved by the institutional Animal Care and Use Committee at Nationwide Children’s Hospital.

### Blood glucose measurements

A drop of blood was drawn from the tail vein and blood glucose concentration was measured using the AlphaTrak veterinary blood glucometer calibrated specifically for rodents (Abbott Laboratories, Abbott Park, IL).

### Preparation of 16 week aortas

Normal heterozygous Db/db (*n* = 4) and type 2 diabetic db/db (*n* = 4) mice were weighed and then euthanized with an overdose of a ketamine/xylazine cocktail (Ketamine, 200 mg/kg and xylazine 20 mg/kg). The chest cavity was cut open and the descending thoracic aorta was bluntly defined from the left subclavian artery to diaphragm. Part of the aortic arch was included for use in cannulation. After flushing the aorta with 20 mL physiological saline solution (PSS, in mmol/L: 130 NaCl, 4 KCl, 1.2 MgSO_4_, 4 NaHCO_3_, 10 HEPES, 1.2 KH_2_PO_4_, 5 glucose, and 2.5 CaCl_2_ at pH 7.4) from the left ventricle, 3 markers were placed on the surface of the descending thoracic aorta using 10–0 monofilament sutures; a calibrated picture was taken to measure the loaded length. The aorta was then extracted and placed in physiological saline for 30 min and another calibrated picture was taken to measure the unloaded length. The vessel was then transferred to the testing laboratory and prepared for mechanical testing.

### Aortic biomechanical testing

The descending thoracic artery was mounted on 700 µm stainless steel cannulae in a biaxial pressure myograph (DMT-USA, Inc. Ann Arbor, Michigan) for mechanical testing as previously described (Lee et al., 2013). Before testing, the unloading length was recorded and the vessel was preconditioned for 3 cycles from 0 to 130 mmHg at the *in vivo* stretch ratio. Three testing cycles were performed consecutively on each vessel: 1) Basal, 2) Active (induced by 100 μmol/L of phenylephrine, and 3) Passive (induced in Ca2+-free PSS by 2 mM EGTA and 100 μmol/L sodium nitroprusside). These doses are known to maximally contract and dilate, respectively, isolated aortic preparations. For each test, the vessel was allowed to achieve stasis after equilibrating it for 10 min at 80 mmHg at the *in vivo* stretch ratio; each testing cycle (basal, active, passive) was then conducted to determine the mechanical properties of the vessel by cycling 3 cycles of pressures from 0 to 130 mmHg. The pressure, force, and outer diameter were simultaneously recorded in all three testing cycles. After *ex vivo* mechanical testing, aortic rings (1–2 mm in length) were cut radially to measure opening. The thickness, inner diameter, and opening angle were measured manually using Image J software.

### Biomechanical data analysis

The thickness was measured *via* calibrated images of the ring segments and the inner diameter was calculated assuming incompressibility. The average circumferential stress, $\sigma_{\theta }$ , was calculated using Eq. 1:

$$\sigma_{\theta }=\frac{Pr_{i}}{r_{o}-r_{i}}$$ (1)

where *p* is the pressure, r_i_ is the changing internal radius and r_o_ is the unloaded inner radius. The average circumferential wall stress, $\lambda_{\theta }$ , was calculated using Eq. 2:

$$\lambda_{\theta }=\frac{r_{middle}}{r_{o,middle}}$$ (2)

where r_middle_ is the central radius and r_o,middle_ is the unloaded central radius. The compliance was calculated as the area change per mmHg for every 10 mmHg pressure step. The incremental elastic modulus in the circumferential direction was calculated as the average change in circumferential stress over stretch ratio for every 10 mmHg pressure step.

### *In Vivo* PWV Experimental Protocol

A separate set of 16 week old mice (Db/db: *n* = 9; db/db: *n* = 5) were instrumented with two pressure-tip catheters under 2% isoflurane anesthesia vaporized with 100% O_2_ (1.2F, SciSense, Transonic Systems Inc. London, Ontario, Canada): One catheter was inserted into the left carotid artery and advanced into the thoracic aorta, and the other was inserted into the left femoral artery and advanced into the abdominal aorta. A microcannula (Fine Science Tools, Inc., Foster City, CA, United States) filled with heparinized 0.9% saline was inserted into the right jugular vein for the administration of drugs during the procedure. All catheters were secured in their respective blood vessels using 5–0 silk.

After instrumentation, mice were allowed to equilibrate for 20–30 min prior to infusing increasing doses of phenylephrine (100–500 nmol/kg/min, Sigma, St. Louis, MO, United States) using a syringe pump (Model 11 Plus, Harvard Apparatus, Holliston, MA, United States) to increase blood pressure. After a washout, mice were then infused with increasing doses of sodium nitroprusside (SNP, 100–600 nmol/kg/min, Sigma, St. Louis, MO, United States) to decrease blood pressure. Care was taken to infuse less than approximately 200 uL of total volume (<10% of approximate circulating blood volume) into each mouse. Blood pressure and heart rate were continuously recorded for the duration of the experiment using LabChart 7 software connected to a PowerLab 16/30 data acquisition system (AD Instruments, Colorado Springs, CO, United States). At the end of the SNP infusion, mice were sacrificed, and the distance between the thoracic and abdominal aorta catheters was measured from 5 to 0 silk that was carefully placed next to the aorta and cut to the length between the two catheters. PWV was calculated as distance between the catheters divided by the time delay between the foot of the pulse waves (reported as cm/ms). PWV was measured at baseline (prior to the administration of any drugs), and at 40–120 mmHg mean arterial pressure during the *in vivo* dose response curves.

### Histology

Perfusion fixed aortas were harvested from 16-wk-old, 24-wk-old, and 36-wk-old normal Db/db (*n* = 4) and diabetic db/db (*n* = 4) mice, fixed in 4% paraformaldehyde in PBS for 24–48 h, and transferred to 70% ethanol until paraffin embedding. Staining for pentachrome was performed according to standard protocols on 16-wk-old samples (Hinton et al., 2006; Levay et al., 2008). Picrosirius red staining was performed as previously described on 16-, 24-, and 36-wk-old samples (Junqueira et al., 1979; Trask et al., 2010). Images were analyzed using ImageJ (NIH) for elastin and collagen expression, which was normalized to medial cross-sectional area (mCSA). Other sections of thoracic aortas from 16-wk-old mice were fixed in 2.5% glutaraldehyde in Millonig’s phosphate buffer for 24–48 h and then stored in Millonig’s buffer until embedding and sectioning. Transmission electron microscopy (TEM) images calibrated and captured using a JEOL JEM-1400 TEM (JEOL Ltd. Tokyo, Japan) equipped with a Veleta digital camera (Olympus Soft Imaging Solutions GmbH, Munster, Germany) as previously described (Tonniges et al., 2016). Aortic collagen fibril diameters (150–550 per animal) were determined by measuring the length of the shortest diameter of collagen cross-sections using ImageJ as previously described (Tonniges et al., 2016).

### Statistical analysis

All data are expressed as mean ± SEM with a probability of *p* < 0.05 used to denote statistical significance using GraphPad Prism 6.0 (GraphPad Software, LaJolla, CA). Two-way repeated measures ANOVA followed by a Bonferroni’s *post hoc* test was performed on pressure myography data. All other measurements were analyzed using an unpaired student’s t-test. Power calculations based on PWV and coronary incremental elastic modulus from Katz et al. (Katz et al., 2011) predicted that *n* = 4 would be required per group to observe potential differences in incremental elastic modulus with greater than 85% power at alpha = 0.05.

## Results

### Body weights and glucose

T2DM db/db mice exhibited higher body weights and blood glucose than non-diabetic Db/db mice (Body weight: Db/db 28.5 ± 1.3 vs. db/db 51.8 ± 1.5 g, *p* < 0.0001; Blood Glucose: Db/db 156 ± 6 vs. db/db 611 ± 7 mg/dL, *p* < 0.0001).

### Aortic diameters and mechanics

**FIGURE 1:**
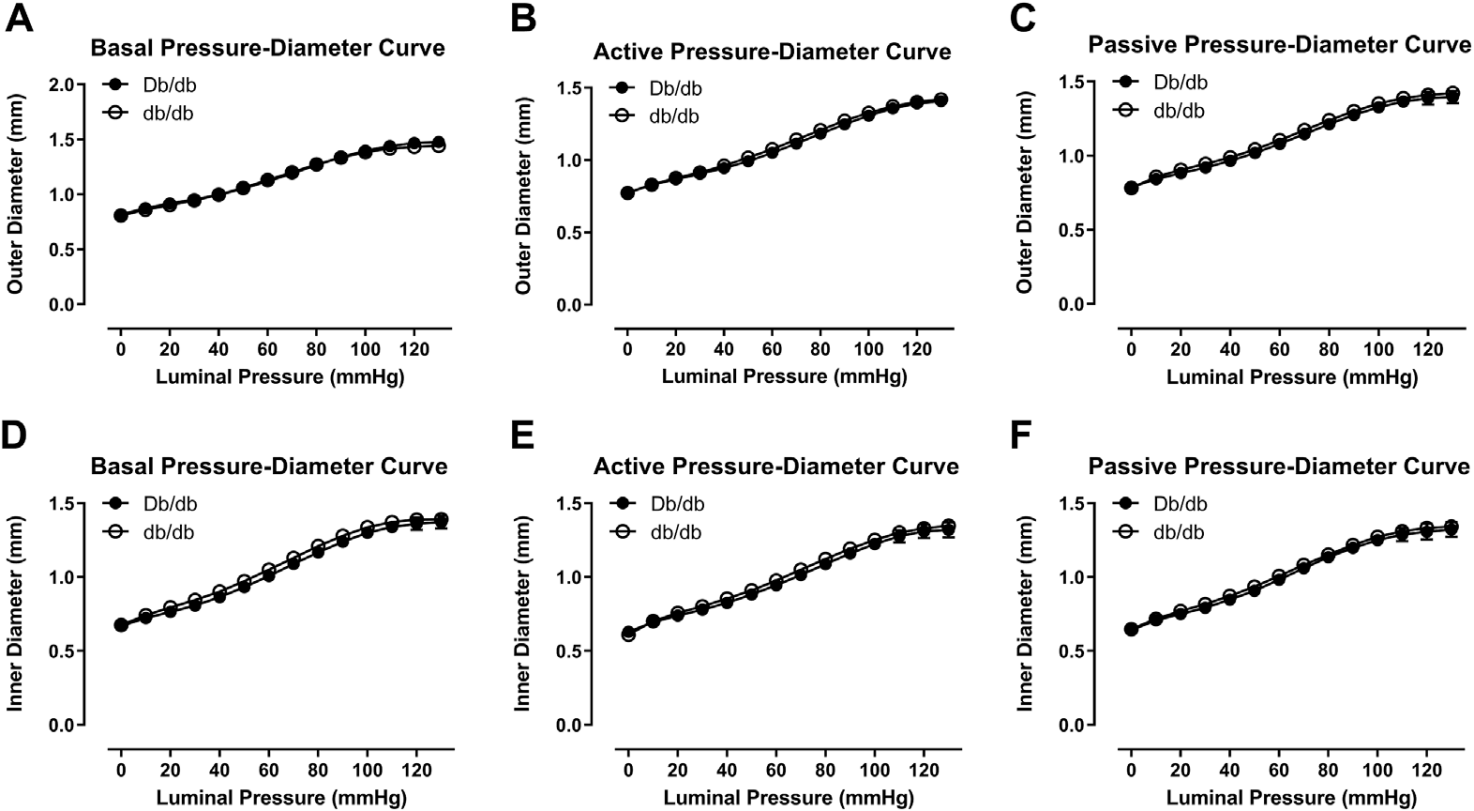
Outer **(A–C)** and inner **(D–F)** pressure-diameter curves were similar between normal and diabetic aortas at 16 weeks of age under basal **(A,D)**, active **(B,E)**, or passive conditions **(C,F)**. *p* = N.S. *n* = 4 per group.

**FIGURE 2:**
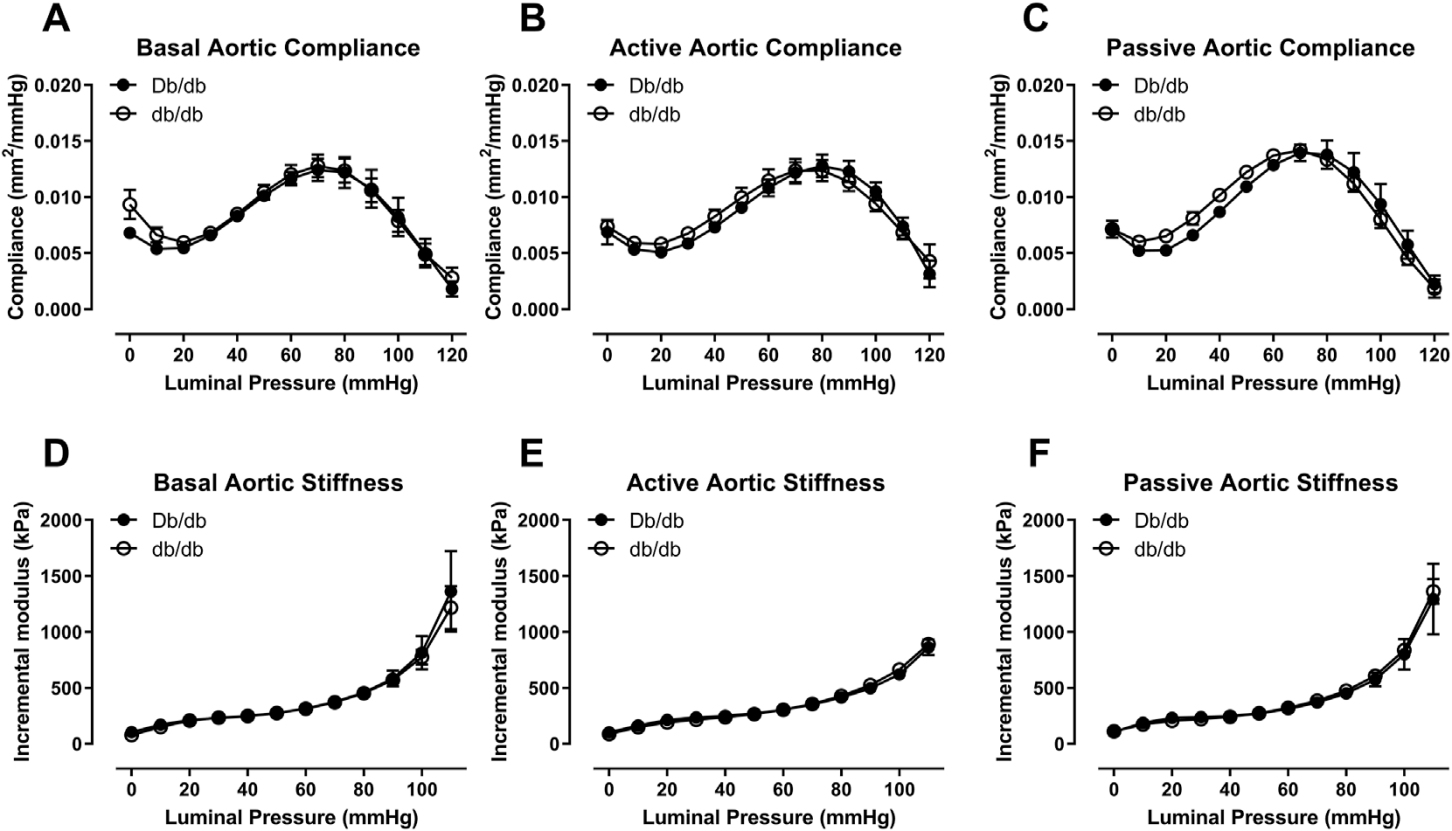
Aortic compliance **(A–C)** and incremental elastic modulus **(D–F)** were similar between normal and diabetic aortas at 16 weeks of age under basal **(A,D)**, active **(B,E)**, or passive conditions **(C,F)**. *p* = N.S. *n* = 4 per group.

In 16 week old mice, biomechanical pressure myography testing revealed no significant differences in outer diameter under basal, active (+Phe), or passive conditions over a range of pressures (Figures 1A–C, *p* > 0.05). Likewise, internal diameters were also unchanged (Figures 1D–F, *p* > 0.05). Wall thickness was unchanged between groups under all three conditions (data not shown). Mechanically, aortas isolated from control and db/db mice exhibited similar vascular compliance (Figures 2A–C, *p* > 0.05) and incremental modulus of elasticity (Figures 2D–F, *p* > 0.05) under basal, active and passive conditions.

### Stretch ratio and histology

**FIGURE 3:**
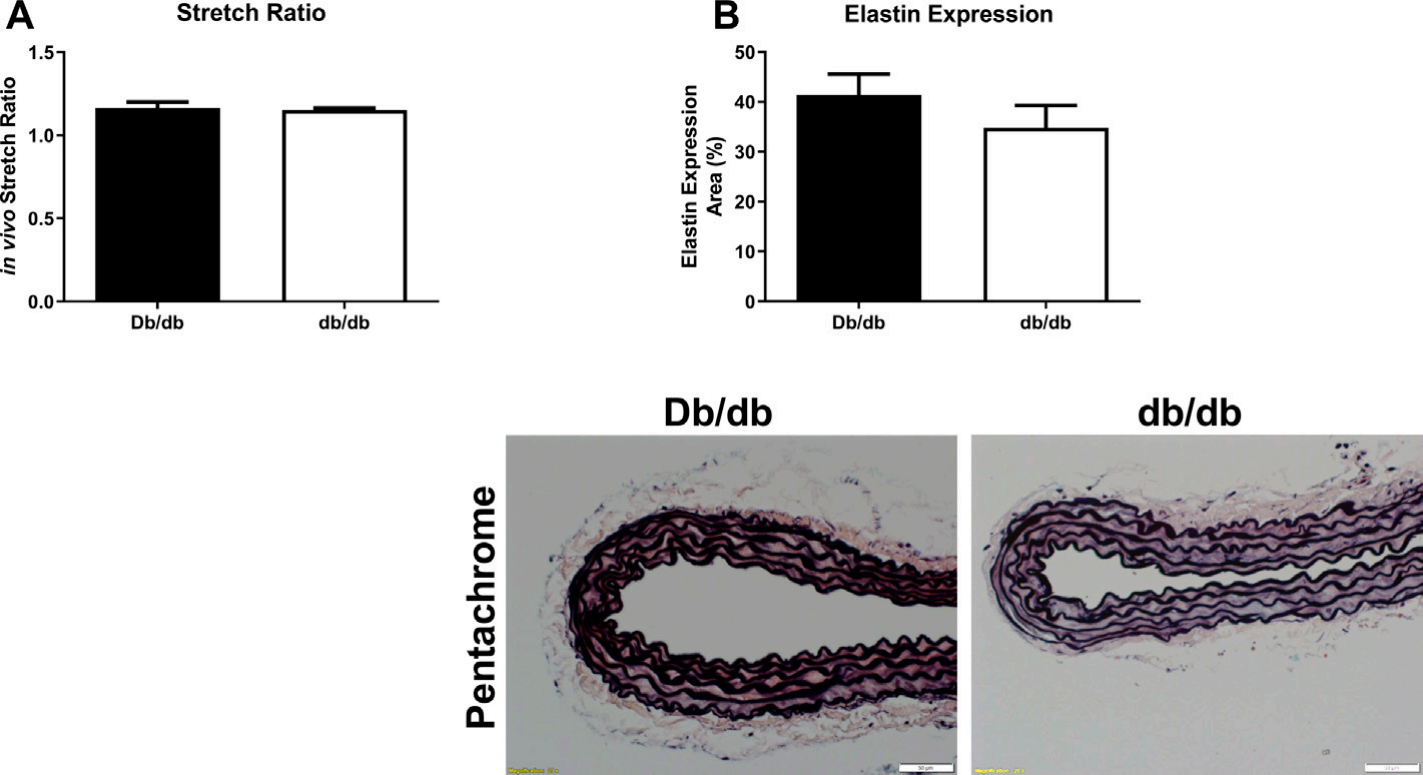
The aortic stretch ratios **(A)** was not different in diabetic db/db mice when compared to Db/db controls. *p* = N.S. *n* = 4 per group. **(B)** Pentachrome staining of normal (top left) and diabetic (top right) aortas revealed no overt differences in elastin staining (black laminar layers), nor did it reveal any appreciable breaks in elastin fiber organization. Scale bars: 50 µm. Representative images from *n* = 4 per group.

**FIGURE 4:**
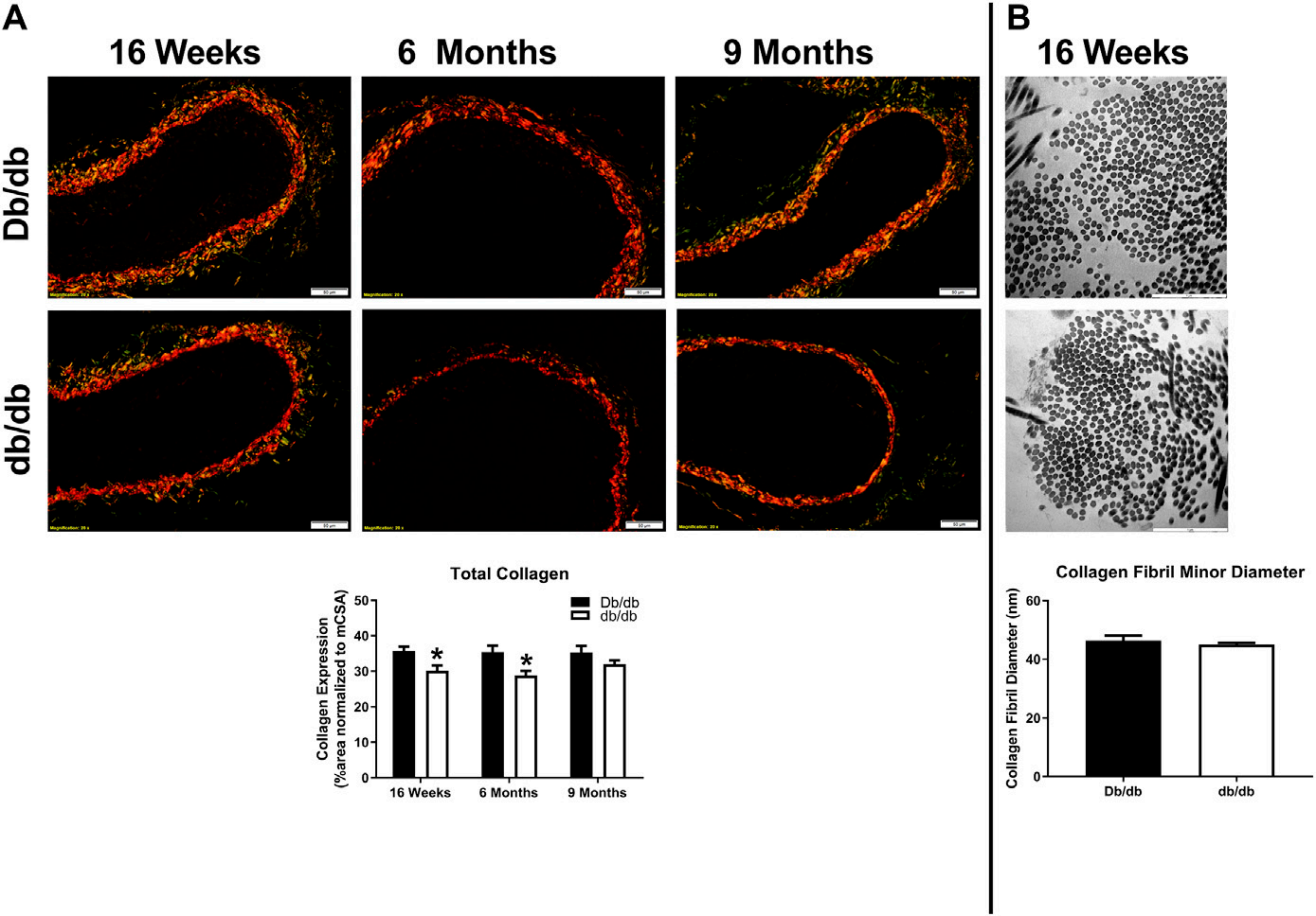
**(A)** Picrosirius red staining of normal and diabetic aortas revealed no overt differences in collagen expression as a function of age (16 weeks–9 months of age). However, collagen expression was significantly lower in diabetic db/db aortas at 16 weeks and 6 months of age. Scale bars: 50 µm. Representative images from *n* = 4 per group. **(B)** Transmission electron microscopy (TEM) images did not reveal differences in collagen fiber diameter between normal and diabetic aortas. Scale bars: 50 µm. Representative images from *n* = 4 per group.

To conduct *ex vivo* experiments at the correct *in vivo* stretch length, we measured aortic stretch ratios. Aortas isolated from T2DM db/db mice exhibited similar stretch ratios compared to control (Figure 3A; Db/db: 1.164 ± 0.037 vs. db/db: 1.154 ± 0.011, *p* > 0.05). Aortic tissues from a separate group of control and db/db mice (*n* = 4–6 per group) were stained by pentachrome and picrosirius red. Pentachrome staining revealed no apparent structural alterations in db/db aortas relative to control, and elastin expression was unchanged (Figure 3B, top, *p* > 0.05). Picrosirius red staining revealed a modest decrease in total collagen detection at 16 weeks and 6 months of age (Figure 4A, *p* < 0.05), which was resolved by 9 months of age (*p* > 0.05). TEM analysis revealed no difference in collagen fibril diameter between normal and diabetic aortas (Figure 4B).

### *In vivo* aortic stiffness by pulse wave velocity

**FIGURE 5:**
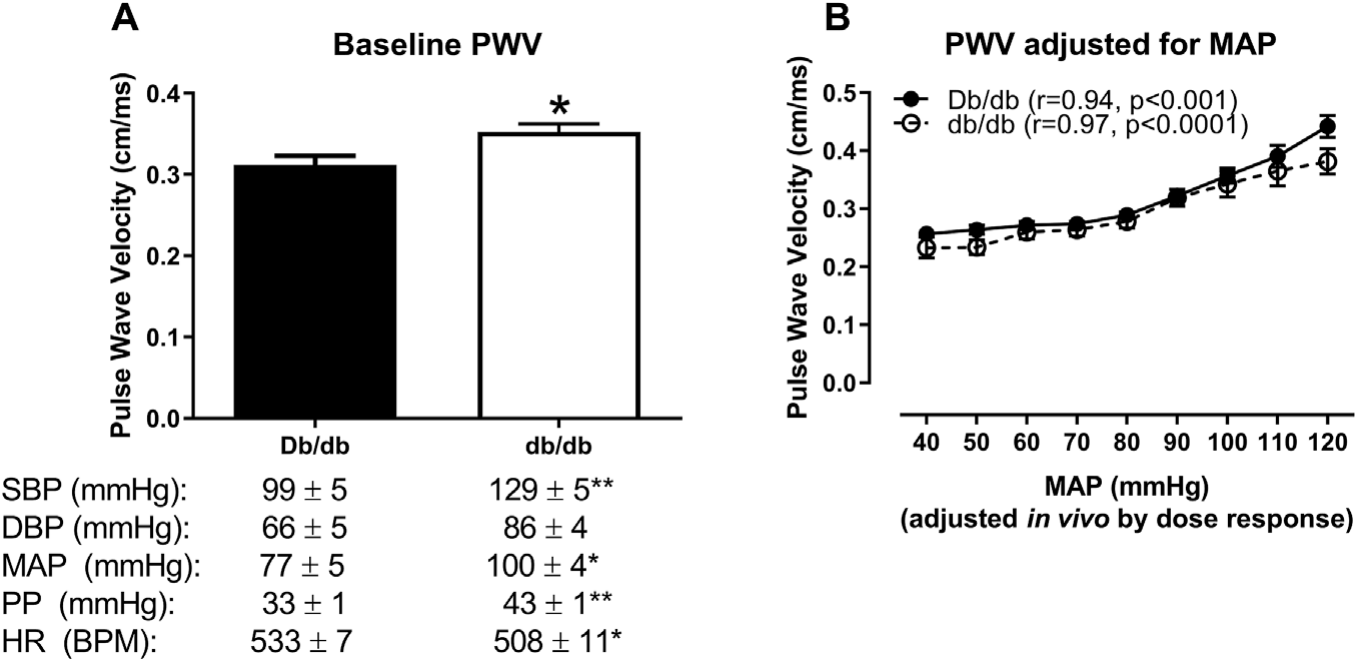
*In vivo* aortic pulse wave velocity measured in anesthetized mice using the gold standard two-catheter method. **(A)** At baseline (measured prior to the infusion of drugs to modulate blood pressure), aortic PWV was augmented in db/db mice, which was associated with an increase in blood pressure and a modest decrease in blood pressure in 16 week-old mice. **(B)** Aortic PWV measured at equivalent mean arterial pressures (MAPs) in response to an *in vivo* dose response to phenylephrine and sodium nitroprusside revealed that the initial increase in db/db aortic PWV was completely abrogated over a range of pressures. *p < 0.05 and **p < 0.01 vs. control Db/db. *n* = 5–9 per group.

To assess whether *in vivo* PWV was dependent upon blood pressure in db/db mice, we conducted an *in vivo* dose response to modulate blood pressure using phenylephrine and sodium nitroprusside. At baseline (prior to the infusion of any drugs), db/db mice showed increased aortic PWV that was associated with a concomitant increase in blood pressure (Figure 5A, Db/db: 0.31 ± 0.01 cm/ms vs. db/db: 0.35 ± 0.01 cm/ms, *p* < 0.05). This increase was completely abrogated when normalized across a range of pharmacologically-modulated equivalent mean arterial pressures (Figure 5B, *p* > 0.05). Furthermore, PWV in both control and diabetic mice significantly correlated with mean arterial pressure (Figure 5B: Db/db: r = 0.94, *p* < 0.001; db/db: r = 0.97, *p* < 0.0001).

## Discussion

Vascular stiffness, classically assessed *in vivo* by pulse wave velocity, is a clinical correlate to adverse cardiovascular diseases and outcomes. For example, increased PWV has been associated with hypertension, diabetes, metabolic syndrome and is a strongly linked to adverse outcomes for myocardial infarction and stroke. Indeed, many of the patient studies that have shown that diabetic conduit arteries have increased stiffness are measured almost exclusively by PWV. In this study, we aimed to test the hypothesis that increased aortic pulse wave velocity in T2DM db/db mice is driven by increased blood pressure, rather than aortic biomechanics. We collected data that showed no difference in *ex vivo* passive stiffness in adult diabetic aortas compared to control. In addition to confirming this finding, here we report that the diabetic aorta does not undergo any overt structural or biomechanical remodeling at 16 weeks of age. We observed no statistical differences in diameters, compliance, incremental elastic modulus, stretch ratio, or elastin expression. Interestingly, we did observe a modest decrease in collagen expression in diabetic aortas that was resolved by 36 weeks of age. Finally, we show here that the increase in diabetic *in vivo* aortic PWV at baseline was completely abrogated when measured at equivalent blood pressures, suggesting that the concomitant increase in blood pressure at baseline accounted for the elevated PWV, and therefore “stiffness.” Collectively, these data may suggest that control of blood pressure, at least in some circumstances, may be sufficient to mitigate the deleterious effects of increased PWV.

The largest confounding variable to pulse wave velocity is mean arterial pressure. (Townsend et al., 2015). Indeed, the dependency of PWV on blood pressure has been previously examined. There are many formulas to calculate PWV in which pressure is a very large variable, again linking a dependency on pressure with changes in PWV. Very early data in the 1920s by McSwiney’s lab (Hickson and McSwiney, 1924) showed that carotid-radial PWV varied with postural blood pressure, suggesting a link between the two. Subsequent studies in the 1950s and 1960s showed that patients with coronary artery disease had higher values of pulse wave velocity than healthy patients (Simonson and Nakagawa, 1960) and PWV was used as early indicator of atherosclerosis in diabetic patients (Simonson and Nakagawa, 1960; Woolam et al., 1962). More recently, Lurbe E et al. report that blood pressure exerts an independent impact on PWV in youth (Lurbe et al., 2012). There is a plethora of more recent data reporting changes in PWV that occur simultaneously with changes in blood pressure in diabetic models and patients (Cruickshank et al., 2002; Kimoto et al., 2003; Sakuragi et al., 2009; Urbina et al., 2010; Isabelle et al., 2016; Omboni et al., 2017). In a recent study, patients given treatment to reduced blood pressure had a significantly lower PWV after treatment (Okon et al., 2016). Stabouli et al. reported that hypertension was linked to increased PWV; however obesity was not a significant determinant in PWV in their study (Stabouli et al., 2015). Given the plethora of studies demonstrating the link between blood pressure and PWV, the role of anti-hypertensive drugs on PWV has been highly studied as well. There is an excellent review by Janic et al. that details the use of various anti-hypertensive drugs and their results altering PWV(Janic et al., 2014). In this review, the use of angiotensin-converting enzyme inhibitors (ACEi), angiotensin receptor blockers (ARBs), beta-blockers, calcium channel blockers reduce blood pressure as well as PWV; however the treatment with nitrates and diuretics appear to have little effect on blood pressure and PWV (Janic et al., 2014). Mechanisms behind this variation in how anti-hypertensive drugs impact PWV include the impact on matrix turnover and the long-term constant of arterial remodeling (Koumaras et al., 2012). Contrary to these previous studies, there have been reports suggesting that an increase in arterial PWV precedes a statistical augmentation in blood pressure. For example, Weisbrod et al. (Weisbrod et al., 2013) showed that non-invasive aortic PWV was increased in male mice after only 1 month on a high fat/high sucrose “western” diet, whereas the elevation in systolic blood pressure did not occur until after 6 months on a western diet. In contrast, DeMarco et al. showed significant elevations in aortic PWV in female mice did not occur until after 4 months on a similar western diet. (DeMarco et al., 2015). Previous data published by our group (Katz et al., 2011; Husarek et al., 2016) showed that while the 24-h average in blood pressure was not different between normal and diabetic db/db mice, it also manifests an elevated blood pressure under isoflurane anesthesia as reflected in the current study. In this study, increased baseline PWV was abrogated through utilization of phenylephrine and sodium nitroprusside to obtain PWV measurements at equivalent MAP. Our current study, combined with numerous previous reports, suggest that an increase in PWV is not necessarily reflective of increased vascular stiffness *per se*, but rather may be secondary to higher blood pressure in certain animal models and populations. Therefore, caution must be exercised when interpreting elevated PWV as a pure indicator of increased vascular stiffness. Here, we show in a controlled animal model, without the confounds of pharmacologic treatment typically associated with human studies, a dissociation of *in vivo* pulse wave velocity and *ex vivo* E_inc_, further emphasizing the importance of considering blood pressure as a confound for PWV changes.

In patient studies, determining aortic stiffness is limited to non-invasive based techniques which is a major reason PWV is so commonly used. There is an excellent review by Laurent et al. that describes the non-invasive techniques for measuring vessel stiffness (Laurent et al., 2006). However, more invasive/terminal techniques may be used in animal models, such as passive pressure myography which was utilized by our laboratory in this study. Utilization of invasive techniques on animal models creates a path for determining true arterial stiffening. Previously, other laboratories have utilized these techniques and reported some aortic biomechanical differences in diabetic and metabolic syndrome animals (Sista et al., 2005; Raaz et al., 2015). Raaz et al. reported that structural aortic stiffening precedes the onset of hypertension in db/db mice at 20 weeks of age, however, they do not report E_inc_; instead, they state diameter differences that are not a conventional method for reporting vessel stiffness (Raaz et al., 2015). Also this group did not show control group myography at the earlier time points so more data may be needed to support their theory (Raaz et al., 2015). In a different study, aortas from Zucker Fatty rats showed an increase in stiffness in the circumferential and longitudinal directions, however statistical values were not reported (Sista et al., 2005). DeMarco et al. reported impaired aortic active stiffness *via* aortic ring data in the presence of calcium and preconstricted with KCl and PWV and aortic endothelial and smooth muscle cell function in untreated Western diet–fed mice (WDC) are improved by mineralocorticoid receptor antagonism, however there were no changes in blood pressure amongst groups (DeMarco et al., 2015). Another group reported that streptozotocin treated diabetic rats showed significantly reduced aortic distensibility and an increased aortic stiffness index (Sun et al., 2009). In a different study, isolated aortic ring segments displayed significantly greater elastic moduli in both young and old SHR *versus* Wistar–Kyoto (WKY) control rats and aging also increased aortic stiffness in this same study (Sehgel et al., 2015). Van Grop et al. reported no difference in elastic modulus between SHR and WKY rats at 1.5 months of age, and interestingly, they reported a decreased incremental modulus in the SHR group at 3 months of age (van Gorp et al., 2000). Overall, there appears to be discrepancies in the hypertensive animal model and the impact of incremental modulus. Vascular stiffness can be influenced by the structural components of the vascular wall (cells, ECM, integrins), as well as by hemodynamics (Sehgel et al., 2015). Numerous papers report PWV and extracellular matrix changes to further support changes in aortic stiffness (Rajzer et al., 2003; Skalska et al., 2005; Paulis et al., 2012). This is a more thorough approach to addressing direct changes in aortic stiffness. Extracellular matrix remodeling underlies age-related aortic stiffening in most models (Lakatta and Levy, 2003; Zieman et al., 2005; Kohn et al., 2015). In studies dating back to the 1960s, it was reported that in normotensive subjects, variations in the velocity of the propagation of the pulse wave are due almost entirely to elasticity alterations (Woolam et al., 1962). It seems that over time, this consideration for the role of blood pressure on PWV is becoming overlooked; however, a recent clinical study reported an association between elevated serum aldosterone concentrations and increased aortic stiffening in normotensive overweight and obese middle aged adults, demonstrating that instances of PWV representing true arterial stiffening in patients may occur (Cooper et al., 2012). Overall, changes in blood pressure should be taken into consideration though when examining the role of PVW as a direct indicator of vascular stiffness.

In summary, despite the db/db mouse model exhibiting many of the known contributors associated with vascular stiffness (hyperglycemia, insulin resistance, mild elevations in blood pressure, hypercholesterolemia, elevated inflammation), elevated PWV in this model remains nearly completely dependent on the distending force of blood pressure and is not a reflection of intrinsic aortic tissue stiffness. Together, these animal model data suggest an intimate regulation of blood pressure during collection of pulse wave velocity when determining *in vivo* vascular stiffness. These data further indicate caution should be exerted when interpreting elevated pulse wave velocity as the pure marker of vascular stiffness.

## Data Availability

The original contributions presented in the study are included in the article/supplementary material, further inquiries can be directed to the corresponding author.
